# ChatGPT With GPT-4 Outperforms Emergency Department Physicians in Diagnostic Accuracy: Retrospective Analysis

**DOI:** 10.2196/56110

**Published:** 2024-07-08

**Authors:** John Michael Hoppe, Matthias K Auer, Anna Strüven, Steffen Massberg, Christopher Stremmel

**Affiliations:** 1 Department of Medicine IV LMU University Hospital Munich Germany; 2 Department of Medicine I LMU University Hospital Munich Germany; 3 Munich Heart Alliance Partner Site Deutsches Zentrum für Herz-Kreislaufforschung (German Centre for Cardiovascular Research) LMU University Hospital Munich Germany

**Keywords:** emergency department, diagnosis, accuracy, artificial intelligence, ChatGPT, internal medicine, AI, natural language processing, NLP, emergency medicine triage, triage, physicians, physician, diagnostic accuracy, OpenAI

## Abstract

**Background:**

OpenAI’s ChatGPT is a pioneering artificial intelligence (AI) in the field of natural language processing, and it holds significant potential in medicine for providing treatment advice. Additionally, recent studies have demonstrated promising results using ChatGPT for emergency medicine triage. However, its diagnostic accuracy in the emergency department (ED) has not yet been evaluated.

**Objective:**

This study compares the diagnostic accuracy of ChatGPT with GPT-3.5 and GPT-4 and primary treating resident physicians in an ED setting.

**Methods:**

Among 100 adults admitted to our ED in January 2023 with internal medicine issues, the diagnostic accuracy was assessed by comparing the diagnoses made by ED resident physicians and those made by ChatGPT with GPT-3.5 or GPT-4 against the final hospital discharge diagnosis, using a point system for grading accuracy.

**Results:**

The study enrolled 100 patients with a median age of 72 (IQR 58.5-82.0) years who were admitted to our internal medicine ED primarily for cardiovascular, endocrine, gastrointestinal, or infectious diseases. GPT-4 outperformed both GPT-3.5 (*P*<.001) and ED resident physicians (*P*=.01) in diagnostic accuracy for internal medicine emergencies. Furthermore, across various disease subgroups, GPT-4 consistently outperformed GPT-3.5 and resident physicians. It demonstrated significant superiority in cardiovascular (GPT-4 vs ED physicians: *P*=.03) and endocrine or gastrointestinal diseases (GPT-4 vs GPT-3.5: *P*=.01). However, in other categories, the differences were not statistically significant.

**Conclusions:**

In this study, which compared the diagnostic accuracy of GPT-3.5, GPT-4, and ED resident physicians against a discharge diagnosis gold standard, GPT-4 outperformed both the resident physicians and its predecessor, GPT-3.5. Despite the retrospective design of the study and its limited sample size, the results underscore the potential of AI as a supportive diagnostic tool in ED settings.

## Introduction

The application of artificial intelligence (AI) has now become part of everyday life. OpenAI has managed to create a highly effective platform with ChatGPT, especially for answering complex questions, positioning it as a pioneer in the field of natural language AI [1]. Despite the emergence of successors such as Google Bard, ChatGPT remains the most widely used platform and was therefore selected for this study.

Especially in the medical field, AI applications offer enormous potential [2]. They can provide helpful guideline-based treatment advice, monitor medication dosages, and signal potential interactions, among other benefits [3]. To date, relevant disadvantages have hardly come to bear. However, the fundamental question of determining reasonable limits for AI applications remains [2].

The first pioneering studies in the field of emergency medicine have just been published. Dahdah and colleagues [4] investigated the capability of GPT-3.5 as a triage tool, noting its ability to provide appropriate responses within a few seconds. Another publication used AI to generate a discharge summary and highlighted the potential benefits of this technology, such as time savings, enhanced accuracy of patient information, and optimized communication [5]. Furthermore, Al-Zaiti and colleagues [6] were able to demonstrate that a machine learning model for electrocardiogram diagnosis of non-ST segment elevation myocardial infarction outperformed both practicing clinicians and other interpretation systems.

The diagnostic accuracy of ChatGPT has, until now, mainly been evaluated using general internal medicine case vignettes, which limits its applicability in a real-world emergency department (ED) setting. Despite using an older version, GPT-3, in their initial study, the authors reported remarkably good performance for the AI chatbot. The accuracy rate for the correct diagnosis among the top 5 differential diagnoses was 98.3% for physicians, compared to 83.3% for GPT-3 (*P*=.03) [7]. In a follow-up study, the same research group reported a diagnostic accuracy slightly above 80% for both physicians and GPT-4 [8].

This study investigates the real-world performance of the latest versions of ChatGPT, specifically those based on GPT-3.5 and GPT-4, regarding their ability to accurately find the right diagnosis in an ED setting when provided with the same information as the treating physician. We performed a blinded head-to-head comparison of the primary treating resident physician versus ChatGPT. The discharge diagnosis, determined after an inpatient stay of several days that included detailed further diagnostics, served as the gold standard.

## Methods

### Assessment of Diagnostic Accuracy

In this retrospective study, we evaluated a cohort of 100 randomly selected adults admitted to our ED in January 2023. The main inclusion criterion was an unplanned inpatient admission due to an internal medical condition. Outpatients and patients presenting with noninternal medical conditions were excluded. The ED resident physician’s diagnosis was defined as the diagnosis documented in the ED letter. Subsequently, each patient’s case history, medical history, current medication regimen, laboratory results, and other diagnostic findings, as documented in the ED letter, were inputted into either GPT-3.5 or GPT-4. The uniform query presented to each chatbot was “What is the most likely diagnosis?” Two examples of representative cases with corresponding input information provided to the chatbot are presented in Multimedia Appendix 1.

Diagnostic accuracy of the ED resident physician and the AI chatbots was then benchmarked against the final hospital discharge diagnosis, which was established after the inpatient stay by senior physicians specialized in the relevant medical fields. Diagnostic performance was ranked on an accuracy point scale of 0 to 2, where 2 points indicated a correct diagnosis, 1 point indicated a partially correct diagnosis, and 0 points denoted an incorrect diagnosis. More specifically, a score of 2 points was awarded for a correct diagnosis that included all major diagnoses identified at admission, regardless of minor diagnoses. A score of 1 point was given for a partially correct diagnosis, which can occur in 2 scenarios: either the major diagnosis is nearly correct and the subtle differences would not have impacted treatment, or there is suspicion of multiple major differential diagnoses, with one being correct and the others incorrect. However, 0 points were assigned when all major diagnoses were incorrect. Minor diagnoses were not scored, since these had no significant impact on the patient’s condition. The term “major diagnoses” refers to the conditions primarily responsible for the patient’s main symptom upon admission to our ED. The grading was performed in a blinded manner by senior physicians trained in emergency medicine (Multimedia Appendix 2).

### Ethical Considerations

Prior to inputting any information into the chatbots, each patient’s data were anonymized and all personally identifiable information was removed according to data privacy standards. The study was performed in accordance with the Helsinki Declaration and was approved by the ethics committee of LMU Munich (23-0445).

### Statistical Analysis

Statistical analyses were performed using Prism (version 9; GraphPad). Ordinal variables were reported as means. For group statistics, we used a 2-way ANOVA with Tukey correction for multiple comparisons. *P* values <.05 were considered statistically significant. No prior sample size calculation was performed.

## Results

### Baseline Characteristics

The median age of our study population was 72 (IQR 58.5-82.0) years, and 45 of our patients were female. The largest proportion, 40 patients in total, were admitted due to cardiovascular diseases. Major pathologies included acute coronary syndrome (n=8), heart failure (n=8), arrhythmias (n=7), and hypertensive crisis (n=4). A total of 22 patients each were admitted with endocrine or gastrointestinal diseases and infectious diseases. The remaining 16 patients presented with kidney or rheumatic diseases (n=9) and pulmonary diseases (n=7) (Table 1, Multimedia Appendix 2).

**Table 1:** Demographics (N=100).

Characteristics			Values
Age (years), median (IQR)			72 (58.5-82.0)
**Sex, n**			
	Male	55	
	Female	45	
**Acute medical condition, n**			
	Cardiovascular diseases	40	
	Acute coronary syndrome	8	
	Heart failure	8	
	Arrhythmia	7	
	Hypertensive crisis	4	
	Pericarditis or myocarditis	2	
	Pulmonary embolism or deep vein thrombosis	2	
	Peripheral artery disease	2	
	Other	7	
**Endocrine and gastrointestinal diseases, n**			
	Total	22	
	Gastrointestinal bleeding	5	
	Diabetes-related complications	4	
	Liver disease	4	
	Cholangitis	3	
	Acute pancreatitis	3	
	Other	3	
**Infectious diseases, n**			
	Total	22	
	Pneumonia	13	
	Urinary tract infection	6	
	Other	3	
**Other diseases, n**			
	Total	16	
	Kidney and rheumatic diseases	9	
	Asthma and chronic obstructive pulmonary disease	7	

### GPT-4 Surpassed GPT-3.5 and Resident Physicians in Diagnostic Accuracy for Emergency Patients

**Figure 1:**
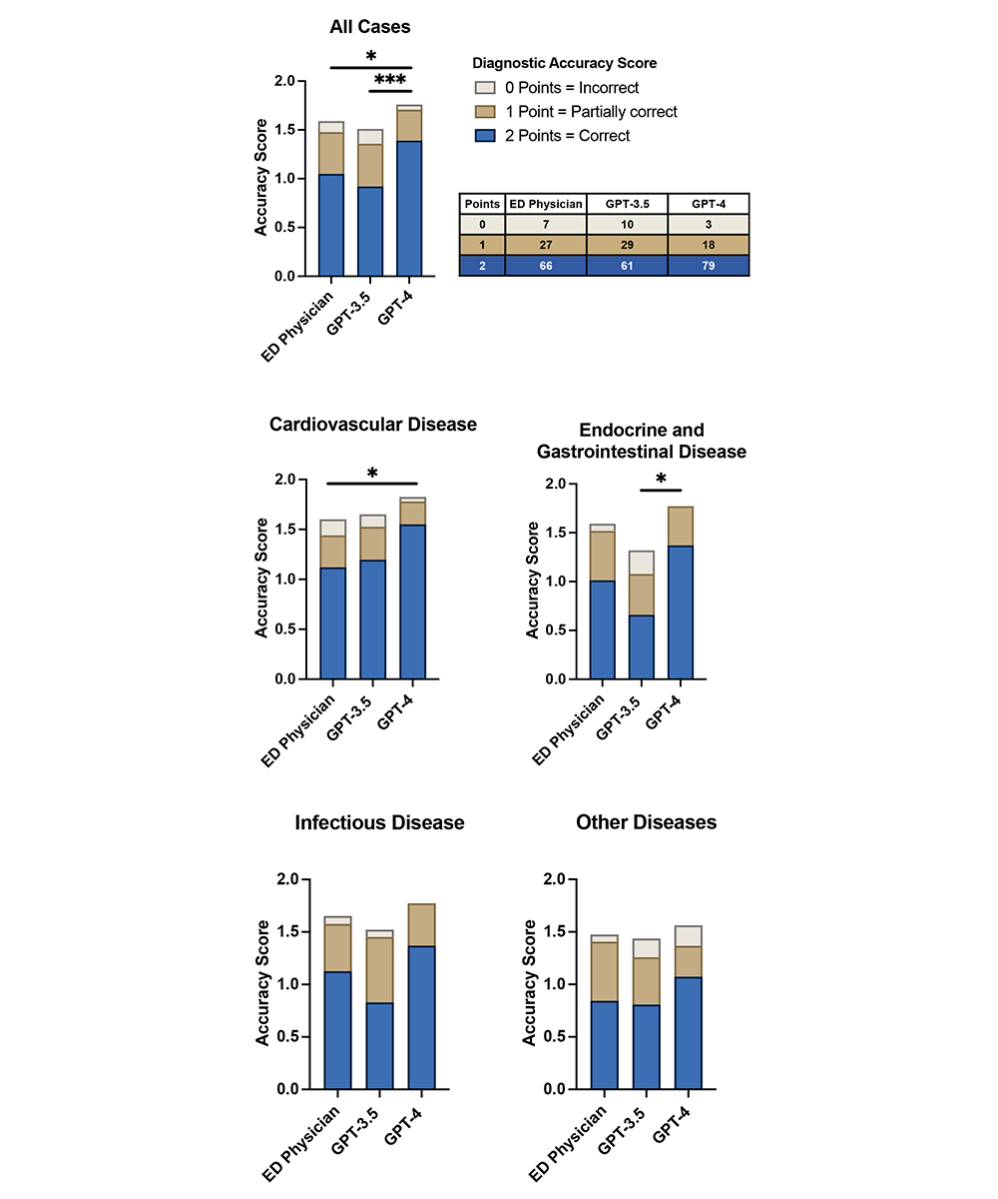
Diagnostic accuracy of resident physicians, GPT-3.5, and GPT-4 for emergency patients. Shown is the mean diagnostic performance across emergency cases (N=100) ranked on a diagnostic accuracy point scale of 0 to 2, where 2 points stand for accurate, 1 point for partially correct, and 0 for incorrect diagnosis. Also, the sample set was stratified in subgroups for cardiovascular diseases (n=40), endocrine and gastrointestinal diseases (n=22), infectious diseases (n=22), and other diseases (n=16). ED: emergency department. *P<.05; **P<.01; ***P<.001.

When comparing GPT-4, GPT-3.5, and resident physicians in predicting the diagnoses of internal medicine emergency patients, GPT-4 demonstrated superior performance (Figure 1). GPT-4 achieved an accuracy score of 1.76 out of a possible 2 points, while GPT-3.5 attained 1.51 points (*P*<.001). Notably, GPT-4 also significantly surpassed the accuracy score of our resident physicians, who achieved a score of 1.59 (*P*=.01). In contrast, the performance of GPT-3.5 was slightly inferior to that of the resident physicians, yet the difference was not statistically significant (*P*=.36).

### GPT-4 Is at Least on Par With or Better Than GPT-3.5 and Resident Physicians in Diagnostic Accuracy Across Multiple Disease Subgroups for Emergency Patients

When stratified for cardiovascular diseases, GPT-4 scored 1.83 points, outperforming GPT-3.5, which scored 1.65, and the resident physician, who scored 1.60. However, only the comparison of GPT-4 versus ED physicians reached statistical significance (*P*=.03) (Figure 1).

For the subgroup of endocrine or gastrointestinal diseases, GPT-4 performed significantly better, with a score of 1.77 points, compared to GPT-3.5 with a score of 1.32 points (*P*=.01). The resident physician achieved 1.59 points, placing them between the 2 performances of the ChatGPT versions, yet the difference compared to GPT-4 was not statistically significant (*P*=.47) (Figure 1).

Within the subgroup of infectious diseases, GPT-4 again outperformed, with a score of 1.77, while GPT-3.5 and the resident physician achieved scores of 1.52 and 1.65, respectively. Compared to GPT-4, these findings did not show a significant difference (Figure 1).

Lastly, we compared the subgroup of “other diseases,” which included kidney and rheumatic diseases, as well as asthma and chronic obstructive pulmonary disease. In the assessment, GPT-4 achieved the highest score at 1.56 points. GPT-3.5 scored 1.44 points and the resident physician 1.50 points. The differences between GPT-4 and the other 2 were not significant (Figure 1).

## Discussion

In this real-world pilot study, we investigated the potential of GPT-3.5 and GPT-4 to identify the correct diagnosis based on patients’ current concerns, medical history, current medication regimen, laboratory results, and other diagnostic findings. We performed a head-to-head comparison of GPT-3.5 versus GPT-4 versus the primary treating resident physician in the ED, evaluating their diagnostic accuracy against the gold standard of the final discharge diagnosis after several days of hospital admission and further examinations.

While GPT-3.5 achieved the same diagnostic accuracy in the overall evaluation, GPT-4 surpassed the ED resident physician. Of note, a direct comparison of GPT-3.5 versus GPT-4 showed a significantly better performance by the latest version—a trend that has already been speculated on in prior evaluations of diagnostic accuracy [7,8]. This superiority of GPT-4 was also evident in the subanalysis of endocrine and gastrointestinal diseases. All other specialty-specific analyses failed to reach statistical significance due to limited case numbers, but showed a trend consistent with the overall cohort findings.

Our observations align with a limited number of smaller previous studies that investigated the diagnostic performance of ChatGPT using clinical vignettes derived from general internal medicine case reports. The rate of correctly identifying a diagnosis among the top 5 suggested differential diagnoses was slightly over 80% for both physicians and ChatGPT [7-9]. In terms of listing a correct or partially correct diagnosis, our real-world study approach reached an accuracy of 93% for the ED physician, 90% for GPT-3.5, and 97% for GPT-4. This high performance likely results from the comprehensive clinical and diagnostic information provided by the treating ED physician. Conversely, ChatGPT performance evaluations that were solely based on the input of self-reported patient symptoms only identified about 50% of the top 3 diagnostic matches [10].

The superiority of ChatGPT lies in its capacity to rapidly generate a range of differential diagnoses, encompassing even rare diseases, thus providing an analytical approach that may surpass the physician. In our study, it is conceivable that the treating resident physician initially considered several differential diagnoses but documented only the most probable one and therefore scored lower. However, it must be assumed that in most cases some differential diagnoses were never considered. This might stem from a physician’s natural tendency to focus on specific symptoms while neglecting subtler ones. Hartigan and colleagues [11] state that physicians are prone to cognitive errors, since both faster intuitive and slower analytical reasoning have potential drawbacks when applied in the clinical setting. When using intuitive reasoning, the physician may unconsciously place a higher weight on personal or patient-specific factors or over- or underemphasize the significance of a data point. Conversely, analytical reasoning is particularly prone to errors in cases where the disease presentation is rare and probability-based decision-making may lead to a more common diagnosis being suspected. GPT-3.5, and particularly GPT-4, due to their analytical data processing, are less prone to some of the errors that can emerge from intuitive reasoning. Example 1 in Multimedia Appendix 1 illustrates a potential cognitive bias among resident physicians. In this instance, a resident assumed that the patient’s chest pain was caused by myocardial infarction, disregarding the patient’s history of lung cancer. This likely resulted from a cognitive error due to fixation on the most probable diagnosis. GPT-3.5 also responded incorrectly, while only GPT-4 identified lung cancer as a possible differential diagnosis.

The retrospective design of our study does not limit the quality of the results, as there was no mutual interference or selection bias. However, the reliance of ChatGPT on information provided by the treating ED resident physician could potentially bias the diagnosis. Additionally, this pioneering study has a limited sample size, which becomes especially apparent in our subanalysis of internal medicine specialties.

Within the broader discussion on the integration of AI in health care, the use of ChatGPT in ED settings raises privacy concerns due to the input of sensitive patient information into systems that may not be entirely secure. There is a risk that this data could be stored or accessed improperly, violating confidentiality laws. Prior to implementing AI in health care settings, it is crucial to ensure the secure management of data.

In the future, AI technologies will become increasingly important in the ED setting, where the time-critical environment demands any supportive tools to facilitate work and improve patient care. ChatGPT and comparable technologies do not compete with resident physicians, but rather assist them in making auxiliary decisions. Moreover, ChatGPT has demonstrated superior diagnostic accuracy in our patient cohort, and future larger studies are needed to confirm this observation and to investigate the use of ChatGPT as a supportive tool in decision-making. We hypothesize that the performance of ChatGPT might even improve with upcoming versions.

## Appendix

Multimedia Appendix 1 Representative cases.

Multimedia Appendix 2 Diagnoses and accuracy scores.

## Abbreviations

AI: artificial intelligence
ED: emergency department
