# Supplementary material for: ChatGPT With GPT-4 Outperforms Emergency Department Physicians in Diagnostic Accuracy: Retrospective Analysis
Source: J Med Internet Res. 2024 Jul 8;26:e56110. doi: 10.2196/56110 (PMC11263899; doi:10.2196/56110)
Supplement: Multimedia Appendix 1 [file jmir_v26i1e56110_app1.docx]

**Example 1 (case #62)**

Case History

Presentation with the emergency service under emergency doctor supervision due to pain in the left shoulder and a feeling of pressure on the left chest since the previous evening. Additionally, she has difficulty breathing deeply. The ECG conducted by the emergency service showed ST-segment depressions in V4-V6. Aspirin 250 mg and Heparin 5000 IU already administered by the emergency doctor.

A single episode of leg thrombosis in the medical history 40 years ago shortly after the birth of her son. No immobilization/surgery in the last 6 months. No hemoptysis. Stool and urination normal, no signs of bleeding.

She lives with her husband at home.

- Allergies: Contrast agent (Imeron 53), house dust mites

Pre-existing Conditions

- NSLCC of the right upper lobe 12/2022
  - Tumor extent: Consolidation dorsobasal right upper lobe 35x30 mm, suspicious pulmonary lesion on the left side
  - Initial tumor stage: cT2a, cN2, cM1a (PUL), UICC Stage IVa
- Pulmonary emphysema
- CT chest: apically accentuated bullous structures
- Coronary artery disease with history of PCI/Stenting in 2013
  - Risk factors: hypertension, hyperlipidemia, nicotine 4-5 cigarettes/day (40 PY)
- History of basal cell carcinoma right lower eyelid
- History of cataract surgery 2021
- History of surgery for skin cancer left lower leg

Medication at Admission

ASS 100 mg 1 - 0 - 0

Candesartan 4 mg 1/2 - 0 - 1/2

Colecalciferol 1000 IE 1 - 0 - 0

Rosuvastatin 20 mg 0 - 0 - 1

Bempedoic acid/Ezetimibe 180/10 mg 0 - 0 - 1

Paracetamol as needed

Physical Examination

General condition slightly reduced, slim body habitus

Heart: Pure and rhythmic tones, no pathological heart sounds

Lungs: Vesicular breathing sounds bilaterally, expiratory wheezing bilaterally, no rales

Abdomen: Soft abdominal wall, no resistances, no acute abdomen, no rebound tenderness, bowel sounds normal in all 4 quadrants

Diagnostic Findings

- ECG: SR, HR 76/min, LAD, PQ 140 ms, QRS 60 ms, QTc 390 ms, normal R progression, no significant disturbance of repolarization
- Echocardiography: Normal left ventricular pump function, no right heart strain, no pericardial effusion, no valvular heart diseases
- Ultrasound: No pleural effusions, no suspicion of pneumothorax, no hydronephrosis bilaterally, no free fluid, vena cava with respiratory modulation, no intra- or extrahepatic cholestasis
- Duplex ultrasound: no deep vein thrombosis in the femoral and popliteal veins bilaterally

Laboratory Findings

Sodium: 136 mmol/l (Reference range: 135 - 145 mmol/l)

Potassium: 4.6 mmol/l (Reference range: 3.5 - 5.1 mmol/l)

Glucose: 95 mg/dl (Reference range: 60 - 99 mg/dl)

Urea: 6 mg/dl (Reference range: 17 - 49 mg/dl)

Creatinine (Jaffé): 1.2 mg/dl (Reference range: 0.5 - 1.0 mg/dl)

GFR (CKD-EPI): 44 ml/min (Reference ≥ 60 ml/min)

Uric Acid: 5.9 mg/dl (Reference range: 2.3 - 6.1 mg/dl)

Calcium: 2.33 mmol/l (Reference range: 2.05 - 2.65 mmol/l)

Albumin-corrected Calcium: 2.28 mmol/l (Reference range: 2.05 - 2.65 mmol/l)

Inorganic Phosphate: 3.0 mg/dl (Reference range: 2.5 - 4.8 mg/dl)

Iron: 156 µg/dl (Reference range: 60 - 180 µg/dl)

Transferrin: 1.9 g/l (Reference range: 2.0 - 3.6 g/l)

Transferrin Saturation: 58% (Reference range: 16 - 45%)

CRP: 5.5 mg/dl (Reference ≤ 0.5 mg/dl)

Procalcitonin: 0.3 ng/ml (Reference ≤ 0.1 ng/ml)

Total Protein: 6.0 g/dl (Reference range: 6.4 - 8.4 g/dl)

Albumin: 4.2 g/dl (Reference range: 3.5 - 5.2 g/dl)

Albumin %: 70.0% (Reference range: 55.8 - 66.1%)

Total Bilirubin: 0.7 mg/dl (Reference ≤ 1.2 mg/dl)

AST [GOT]: 33 U/l (Reference ≤ 34 U/l)

Gamma-GT: 20 U/l (Reference ≤ 39 U/l)

LDH: 220 U/l (Reference ≤ 249 U/l)

Creatine Kinase (CK): 43 U/l (Reference ≤ 169 U/l)

CK-MB Activity: 20 U/l (Reference ≤ 24 U/l)

Troponin T (hs): 0.025 - 0.032 ng/ml (Reference ≤ 0.014 ng/ml)

Cholesterol: 149 mg/dl (Reference < 200 mg/dl)

Triglycerides: 116 mg/dl (Reference < 150 mg/dl)

LDL-Cholesterol: 43 mg/dl (Reference < 116 mg/dl)

HDL-Cholesterol: 85 mg/dl (Reference > 48 mg/dl)

Non-HDL-Cholesterol: 64 mg/dl (Reference < 146 mg/dl)

Bicarbonate: 24 mmol/l (Reference range: 21 - 31 mmol/l)

Erythrocytes: 3.62 T/l (Reference range: 3.96 - 5.16 T/l)

Hemoglobin: 12.1 g/dl (Reference range: 11.5 - 15.4 g/dl)

Hematocrit: 0.345 l/l (Reference range: 0.346 - 0.453 l/l)

MCV: 95.3 fl (Reference range: 80.0 - 95.5 fl)

MCH: 33.4 pg (Reference range: 26.1 - 32.6 pg)

MCHC: 35.1 g/dl (Reference range: 31.9 - 35.5 g/dl)

Platelets: 165 G/l (Reference range: 176 - 391 G/l)

Normoblasts (Automated): < 0.1 /100 Leu (Reference ≤ 1.0)

RDW-CV: 11.9% (Reference range: 12.1 - 14.8%)

PDW: 13 fl (Reference range: 10 - 15 fl)

MPV: 11.2 fl (Reference range: 9.2 - 12.5 fl)

Neutrophil Granulocytes: 80% (Reference range: 43 - 74%)

Lymphocytes: 18% (Reference range: 18 - 46%)

Monocytes: 2% (Reference range: 4 - 12%)

Eosinophil Granulocytes: < 1% (Reference ≤ 5%)

Basophil Granulocytes: < 1% (Reference ≤ 1%)

Neutrophil Granulocytes: 8.42 G/l (Reference range: 1.91 - 7.37 G/l)

Lymphocytes: 1.86 G/l (Reference range: 1.22 - 3.56 G/l)

Monocytes: 0.16 G/l (Reference range: 0.25 - 0.85 G/l)

Eosinophil Granulocytes: 0.07 G/l (Reference range: 0.03 - 0.44 G/l)

Basophil Granulocytes: 0.02 G/l (Reference range: 0.01 - 0.08 G/l)

Quick Value: 66 - 44% (Reference ≥ 70%)

INR: 1.3 (Reference range: 0.8 - 1.2)

aPTT (LA-sensitive): >180 sec (Reference range: 25 - 42 sec)

Fibrinogen (Clauss): 242 mg/dl (Reference range: 160 - 400 mg/dl)

TSH: 0.85 µU/ml (Reference range: 0.27 - 4.20 µU/ml)

Folate: >20.0 ng/ml (Reference range: 5.6 - 45.8 ng/ml)

Vitamin B12: 1552 pg/ml (Reference range: 197 - 771 pg/ml)

NT-proBNP: 730 pg/ml (Reference ≤ 738.0 pg/ml)

Urine analysis:

Sodium (Urine): 101 mmol/l (Reference range: 64 - 172 mmol/l)

Sodium/Creatinine Ratio (Urine): 228 mmol/g (Reference range: 54 - 360 mmol/g)

Urea (Urine): 12.3 g/l (Reference range: 17.00 - 36.00 g/l)

Urea/Creatinine Ratio (Urine): 27.8 g/g (Reference ≤ 12.00 g/g)

Creatinine (Urine): 44.3 mg/dl (Reference range: 29.0 - 226.0 mg/dl)

Total Protein (Urine): 9.0 mg/dl (Reference ≤ 15.0 mg/dl)

Protein/Creatinine Ratio (Urine): 203 mg/g (Reference ≤ 100.0 mg/g)

Albumin (Urine): 3.1 mg/dl (Reference ≤ 2.0 mg/dl)

Albumin/Creatinine Ratio (Urine): 70.0 mg/g (Reference ≤ 20.00 mg/g)

Alpha-1-Microglobulin (Urine): 26.0 mg/l (Reference ≤ 12.0 mg/l)

Alpha-1-Microglobulin/Creatinine Ratio (Urine): 58.7 mg/g (Reference ≤ 20.00 mg/g)

Urine test strip:

Blood: Negative, Leukocytes: Approx. 15 /µl (Negative), Nitrite: Negative, pH: 5.5 (Reference range: 5.0 - 7.0), Protein: Negative, Glucose: Negative, Ketone Bodies: Negative, Urobilinogen: 0.2 mg/dl (Reference ≤1.0), Bilirubin: Negative, Specific Gravity: 1010 g/l (Reference range: 1015 - 1025)

Urinary sediment:

Leukocytes: 3-10 /Field (Reference ≤ 5), Squamous Epithelial Cells: Present, Leukocytes: 6.80 G/l (Reference range: 4.00 - 10.40 G/l)

**Example 2 (case #95)**

Case History

Sudden onset of abdominal pain, nausea, and vomiting. Initially vomited food remnants, then bile. Cramp-like upper abdominal/epigastric pain. After administration of Pantoprazole and Dimenhydrinate, there was a significant improvement in symptoms. The patient attributes the complaints to eating spoiled meat. Self-administration of Paracetamol and Pantoprazole.

- Nicotine: no
- Alcohol: not recently, before that 1 glass of beer per day
- Allergies: Grasses

Pre-existing Conditions

- Atrial fibrillation
- Heart failure EF 40%, Dilated cardiomyopathy
- 2021 Cardiac catheter exclusion of CHD
- Kidney failure (some operation years ago? Abscess? Kidney sand?)
- Gallstones
- Constipation
- COPD?

Medication at Admission

Torasemide 20 mg 1 - 0 - 1

Apixaban 5 mg 1 - 0 - 1

Bisoprolol 2.5 mg 1 - 0 - 0

Simvastatin 20 mg 0 - 0 - 1

Candesartan 8 mg 1 - 0 - 0

Spironolactone 25 mg 1 - 0 - 0

Dulcolax 2.5 mg 0 - 0 - 2

Sodium bicarbonate in the evening

Foster as needed

Pantoprazole 40 mg as needed

Physical Examination

Heart: Heart sounds quiet

Lungs: bilaterally vesicular breath sounds

Abdomen: soft, significant tenderness in the epigastric region, no flank percussion pain, no guarding, quiet bowel sounds

Diagnostic Findings

Ultrasound: enlarged gallbladder without tri-layering, cholecystolithiasis.

Laboratory Findings

Hyperkalemia 5.5 mmol/l (in control 4.8 mmol/l)

Creatinine 2.0 mg/dl

GFR 34 ml/min

GOT 100 U/l

GPT 82 U/l

gamma-GT 78 U/l

Lipase >3000 U/l

Troponin 0.057 in control 0.046 ng/ml

NT-pro BNP 1158 pg/ml

no CRP elevation

no leukocytosis

normal LDH

Hct 0.368

Blood gas analysis: pH 7.3, Lactate 1.7 mmol/l, K 4.8 mmol/l
